# Supplementary material for: Evidence supported by Mendelian randomization: impact on inflammatory factors in knee osteoarthritis
Source: Front Med (Lausanne). 2024 May 28;11:1382836. doi: 10.3389/fmed.2024.1382836 (PMC11165061; doi:10.3389/fmed.2024.1382836)
Supplement: Supplementary file 1 [file Data_Sheet_1.ZIP › data/GCST90274807_5e6/807.leaveoneout.pdf]

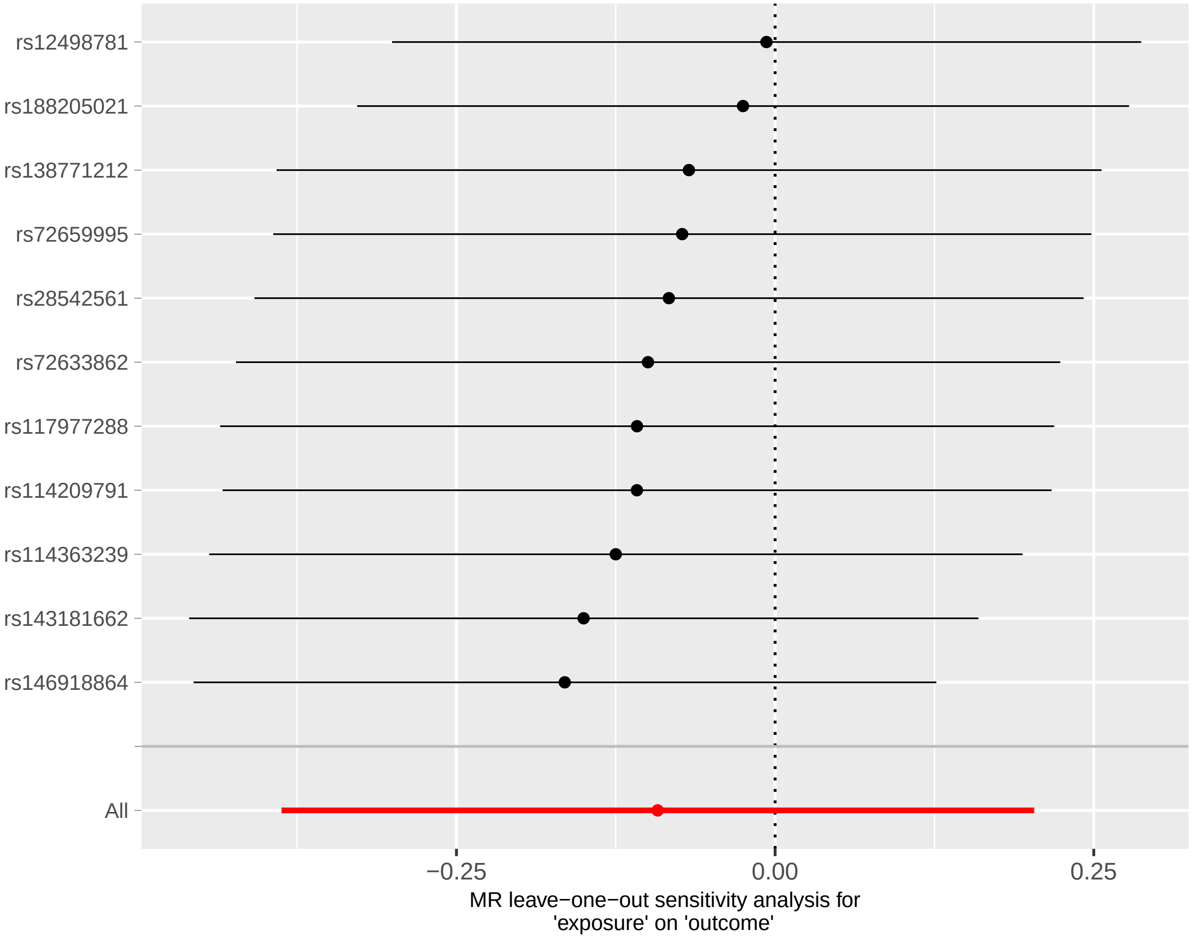
rs12498781
rs188205021
rs138771212
rs72659995
rs28542561
rs72633862
rs117977288
rs114209791
rs114363239
rs143181662
rs146918864
All
−0.25
0.00
0.25
MR leave−one−out sensitivity analysis for
'exposure' on 'outcome'
